# New Appliances and Things Medical

**Published:** 1897-04-24

**Authors:** 


### NEW APPLIANCES AND THINGS MEDICAL.

### FIRST SWISS BRAND CONDENSED MILK.

(Alpine Milk Exporting Co., 3, Laurence Pountney Hill, London, E.C.)

This is a really genuine condensed milk without any added sugar or preservatives, and its degree of concentration is not over-exaggerated by the vendors. The analysis shows that it is obtained by evaporating genuine cow's milk, not skim milk as is occasionally the case, to about a third of its bulk, and then sealing in tins. As it contains no preservative, the milk should be used in the same way as ordinary milk, after the addition of two or three parts of water—not four as stated by the company, which would make it equivalent to a milk containing 10 to 20 per cent. of added water. Our analysis agrees very closely with those published by the vendors, and is as follows:—

| | |
|---|---|
| Fat | 11·05 |
| Milk Sugar | 13·85 |
| Casein and Albumen | 10·18 |
| Mineral Matter | 2·09 |
| Cane Sugar | None |
| Water | 62·83 |
| | 100·00 |

We cannot too strongly advocate the use of such condensed sterilised milk, but hope that all our readers will warn their friends and patients as to the importance of reading the label, and seeing that the tin contains either this particular brand or one which agrees with it in containing pure milk, unskimmed, and free from cane sugar and preservatives.

